# Lack of plasma albumin impairs intravascular lipolysis and explains the associated free fatty acids deficiency and hypertriglyceridemia

**DOI:** 10.1186/1476-511X-9-146

**Published:** 2010-12-27

**Authors:** Tiago R Figueira, Aníbal E Vercesi, Helena CF Oliveira

**Affiliations:** 1 Departamento de Patologia Clínica, Faculdade de Ciências Médicas, UNICAMP - Universidade Estadual de Campinas, Campinas, SP, Brazil; 2 Departamento de Fisiologia e Biofísica, Instituto de Biologia, UNICAMP - Universidade Estadual de Campinas, Campinas, SP, Brazil

## Abstract

**Background:**

Abnormalities in lipid metabolism and transport are hallmarks in analbuminemic Nagase rats (NAR) and humans. Triglyceridemia is nearly 3- to 5-fold higher in female NAR than in control Sprague-Dawley rats (SDR). Also, NAR present with a severe plasma free fatty acid (FFA) deficit. There are conflicting results regarding the mechanisms underlying NAR hypertriglyceridemia.

**Objective:**

We aimed at investigating whether liver lipogenesis and triglyceride secretion rates into the plasma contribute to the hypertriglyceridemia in NAR. We also studied whether heparin or albumin administration would release the hypothesized lipolysis inhibition in NAR.

**Methods:**

The incorporation of tritiated water into lipids and the linear accumulation rate of plasma triglycerides after Triton WR1339 injection were the measures of liver lipogenesis and triglyceride secretion rates.

**Results:**

Lipogenesis (596 ± 40 vs. 929 ± 124 μmol ^3^H_2_O/g/h) and triglyceride (4.25 ± 1.00 vs. 7.04 ± 1.68 mg/dL/min) secretion rates were slower (*P *≤ 0.05) in fasted NAR than in control SDR. The injection of either heparin or albumin elicited an increase in NAR plasma FFA levels over time. FFA levels reached control levels 90 min after the albumin administration, increasing from 0.36 ± 0.05 to 1.34 ± 0.16 mEq/L (*P *≤ 0.05). These results indicate that the lack of plasma albumin inhibits intravascular lipolysis and causes the FFA deficit observed in NAR.

**Conclusion:**

NAR hepatic triglyceride synthesis and output do not contribute to NAR hypertriglyceridemia. We propose that the lack of albumin diminishes intravascular lipolysis which reduces the plasma triglyceride removal rate and explain both NAR hypertriglyceridemia and FFA deficiency.

## Introduction

Albumin is the most abundant plasma protein in mammals and plays an important role as a carrier for a variety of molecules. Albumin possesses a high binding affinity for certain divalent cations, bilirubin, free fatty acids (FFA) and other molecules, including xenobiotics. Due to its high abundance and low molecular weight in relation to other major plasma proteins, albumin is responsible for about 80% of the total plasma oncotic pressure [1]. Therefore, albumin is a key regulator of fluid distribution between the plasma and interstitial compartments in physiological conditions; although the absence of plasma albumin can be well compensated by increased liver secretion of other proteins which help to maintain nearly normal plasma oncotic pressure [2]. Other less understood functions may also be ascribed to albumin, since its plasma depletion and redox modification have been demonstrated in several pathological conditions [3-5].

Both humans and rats (Nagase rats, NAR) suffering from congenital analbuminemia present with minor clinical symptoms despite the ascribed roles of albumin [6-8]. Abnormalities in fat storage and in lipid metabolism and/or transport are hallmarks of analbuminemic NAR and humans [9-15]. Plasma triglyceride (TG) levels are 3- to 5-fold higher in female NAR than in control Sprague-Dawley rats (SDR). A more severe dyslipidemia in female NAR seems to be partially driven by estrogen [13]. Compared to male, female NAR also develop more efficient adaptations to the lack of plasma albumin and do not present a deficit in total plasma proteins [2]. In analbuminemic plasma, very low density lipoprotein triglycerides (VLDL-TG) accounts for the largest fraction of total triglycerides [15]. Conversely, plasma FFA levels are severely decreased in NAR compared to control [14]. In addition, analbuminemic rats and human subjects present with hypercholesterolemia [6,9,11,12]. This lipemic phenotype predisposes the affected individuals to a higher risk of developing cardiovascular disease, while low levels of FFA may contribute to other metabolic-related alterations, such as the fatigue experienced by analbuminemic individuals [7,11,12,16].

Studies addressing the basis of hypertriglyceridemia in NAR have shown conflicting results. Catanozzi et al. [17] reported a faster triglyceride secretion rate from the liver and a slightly faster VLDL-protein plasma clearance rate. In contrast, others have demonstrated that NAR present with a slower plasma clearance rate of chylomicron-TG [16] or a similar clearance rate of VLDL- and chylomicron-TG [18]. In addition, lower post-heparin plasma lipoprotein lipase (LPL) activity has been described in NAR [13,19]. Regarding the hepatic production of triglycerides, Joles et al. [9] showed that the liver lipogenesis rate is not different between NAR and SDR. Therefore, no clear conclusions can be drawn concerning the main metabolic processes contributing to NAR hypertriglyceridemia. Differences in criteria for animal group pairing (by body mass or by age), sex, basal states (fasted vs. fed) and methodology may account for these data discrepancies.

Despite the known roles of albumin as a FFA acceptor and carrier [20], there are no data on the mechanism underlining the low levels of plasma FFA found in NAR. It is still unknown whether the FFA deficit is a consequence of lipolysis inhibition, either directly by lower LPL activity [13,19] or by a deficiency in plasma albumin, or by both. Abnormalities, such as lower body mass and adiposity [10] and the exercise and starvation intolerance observed in NAR [21,22], seem consistent with a condition of lower FFA availability and flux into tissues [16,23].

In this work, we found that liver lipogenesis and hepatic triglyceride output are decreased in fasted NAR compared to control. We also show that the low levels of plasma FFA are due to an inhibition of intravascular lipolysis caused by an acceptor (albumin) deficiency in NAR. All together, our data indicate that hepatic triglyceride production does not contribute to NAR fasting hypertriglyceridemia.

## Materials and methods

### Animal housing and plasma variable assessments

NAR founders were kindly donated by Dr. Eder Quintão from the Lipid Laboratory at the University of São Paulo Medical School and were bred and maintained in the animal facility of our department. The experiments were approved by the Committee for Ethics in Animal Experimentation at the University and are in accordance with the Guide for the Care and Use of Laboratory Animals published by the National Academy of Sciences. The rats had free access to a standard laboratory rodent chow diet (Nuvital CR1) and water. Female rats, 12-14 weeks old, were housed at 22 ± 2°C on a 12 h light-dark cycle. Unless otherwise stated, all the measurements were carried out in female NAR after a 20-h fasting, and the blood samples were taken from the tip of the tail. Triglycerides (Roche Diagnostics), total cholesterol (Roche Diagnostics), total proteins (Bradford, Sigma) and free fatty acids (Wako Chemicals, Japan) were determined in the plasma using enzymatic-colorimetric methods according to the instructions of the manufacturers.

### Total liver lipogenesis

The rate of total lipid synthesis was measured *in vivo*. The rats were injected intraperitoneally with 20 mCi of tritiated water (^3^H_2_O) dissolved in an isotonic saline solution as described previously [24]. One hour later, blood samples were obtained from the tip of the tail from anesthetized rats (ketamine 75 mg/kg and xylazine 10 mg/kg), and livers were excised, minced, and saponified; after which lipids were extracted with hexane [25]. Radioactivity in the lipid extract was measured in a Beta Counter (LS6000 Beckman Instruments, California, USA). The specific activity of ^3^H_2_O was measured in the plasma in triplicate. The rate of total lipid synthesis was calculated as μmol of ^3^H_2_O incorporated into the lipids per gram of tissue in one hour (μmol/g/h).

### Triglyceride secretion rate

The hepatic triglyceride secretion rate to the plasma was measured after the administration of Tyloxapol WR1339 (Sigma), as described previously by Otway & Robinson [26]. Briefly, rats were bled to obtain baseline plasma samples and then injected intravenously (tail vein) with Tyloxapol WR1339 at a dose of 400 mg/kg (15% v/v solution in saline). Blood samples (50 μL) were collected at 15, 30, 60, and 90 min after the Tyloxapol WR1339 injection. Triglyceride levels were measured in the plasma samples. The slopes from the linear regression of the triglyceride concentrations vs. time curves were calculated (mg/dL/min). Experiments were performed between 10:00 and 13:00 h. Additional groups of overnight sucrose-fed (5% in drinking water) female and male NAR were also subjected to this experiment. These rats were fed with carbohydrate only to avoid interference of absorbed fat.

### Albumin- or heparin-stimulated *in vivo *lipolysis

Sodium heparin (Cristália, Brazil) and bovine serum albumin (globulin and fatty acid free, Sigma) were diluted in saline. The rats were injected with heparin (1,000 U/Kg, intraperitoneally) or with albumin (1.3 g/kg, intravenously). Blood samples were taken before (0 min) and after the injections (30 and 90 min). After blood plasma was separated by centrifugation (4°C, 3000 g for 10 min), plasma FFA levels were measured. FFA levels were also measured in an aliquot of the injected albumin solution but were not detected. In order to control for likely changes in plasma volume due to the intravascular albumin injection (0.27 mL per 100 g), additional NAR and SDR groups were injected with saline at the same volume. The amount of injected albumin (1.3 g/kg) was expected to elevate plasma albumin concentrations from nearly zero to above 17 mg/mL in NAR [17]. As conducted in females, the lipolysis induced by intravenous albumin injection was also studied in fasting male NAR, but plasma free glycerol levels, instead of plasma FFA, were measured enzymatically (Sigma).

### *In vitro *serum lipolysis

Blood samples from resting rats were taken without anti-clogging agents and immediately centrifuged (4°C, 3000 g for 10 min) to separate the serum. Aliquots of the serum (120 μL) were incubated with exogenous albumin (final concentration of 30 mg/mL) or its vehicle (saline) at 37°C for 10 min with continuous shaking. Aliquots (20 μL) were withdrawn and quickly frozen in liquid nitrogen for later FFA analysis. Lipolysis was calculated as an increase in FFA concentration between the time points (zero to 10 min).

### Statistical analysis

Data are represented as mean ± SD. Differences between means were assessed by a Student's t-test or one- or two-way analysis of variance (ANOVA) with repeated measures. A one-way ANOVA was run within the NAR albumin group to test for the effects of the albumin injection (Figure 2A) because we compared the pre-and post-injection values within this group only. The significance level was set at *P *≤ 0.05.

## Results

The body and liver masses and the concentrations of the plasma lipids and proteins from NAR and SDR are shown in Table 1. As compared to SDR of the same age, 12-14 week old female NAR exhibited lower body and higher liver masses, a 5-fold increase in TG levels, a 2.6-fold increase in cholesterol levels, and one-third of the FFA concentrations (*P *≤ 0.05). Enlarged NAR livers probably reflect hypertrophy to compensate for the albumin deficiency, leading to additional synthesis and secretion of other plasma proteins.

**Table 1:** Body and liver masses and plasma lipid and protein concentrations in analbuminemic (NAR) and control (SDR) rats.

	SDR	NAR
Body Mass (g)	285.8 ± 24.8 N = 9	216.4 ± 8.7* N = 9
Liver Mass (g/100 g)	3.4 ± 0.2 N = 9	5.5 ± 0.4* N = 9
Plasma Proteins (g/L)	59.3 ± 8.0 N = 4	55.7 ± 3.3 N = 4
Triglycerides (mg/dL)	62.2 ± 20.1 N = 10	316.8 ± 99.8* N = 10
Cholesterol (mg/dL)	72.6 ± 15.5 N = 10	189.2 ± 22.8* N = 10
FFA (mEq/L)	1.53 ± 0,22 N = 8	0.48 ± 0,06* N = 8

Mean ± SD. *P ≤ 0.05 vs. SDR. Female Nagase rats (NAR) and Sprague-Dawley rats (SDR) were paired by age (12 to 14 weeks old). Liver mass expressed as gram per 100 g of total body mass. FFA: free fatty acids.

**Figure 1:**
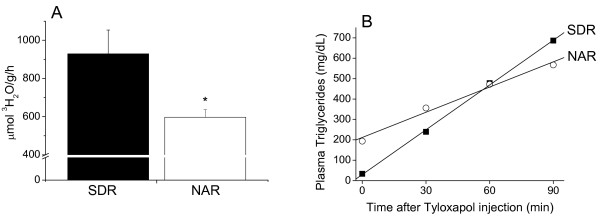
**Total liver lipogenesis rate and triglyceride secretion rate into the plasma**. A. Mean ± SD of tritiated water incorporation into total lipids after one hour of ^3^H_2_O injection. N = 4 in each group. B. Representative curves of plasma triglyceride response to Tyloxapol WR1339 injection. After the Tyloxapol WR1339 inhibition of triglyceride lipolysis, the rate of plasma triglyceride accumulation was taken as the measure of triglyceride secretion into the plasma. The mean ± SD of the triglyceride secretion rates were 7.04 ± 1.68 and 4.25 ± 1.00 mg/dL/min (*P *≤ 0.05) for SDR and NAR, respectively. N = 5 in each group. *P ≤ 0.05.

The liver lipogenesis rate (596 ± 40 vs. 929 ± 124 μmol ^3^H_2_O/g/h) and the hepatic TG secretion rate into the plasma (4.25 ± 1.00 vs. 7.04 ± 1.68 mg/dL/min) were significantly (*P *≤ 0.05) reduced in the 20-h fasted NAR compared to the SDR controls (Figure 1). In overnight sucrose-fed female (n = 4 each group) and male (n = 6 each group) rats, the hepatic TG secretion rates into the plasma were not significantly different between NAR and SDR: 5.27 ± 0.73 vs 6.08 ± 0.56 mg/dL/min for females, and 2.95 ± 0.77 vs 3.67 ± 0.82 mg/dL/min for males. Therefore, irrespective of feeding state and sex, liver triglyceride output does not explain the higher TG plasma levels in NAR.

**Figure 2:**
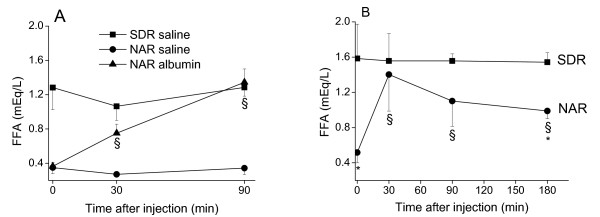
**Plasma free fatty acid (FFA) response to albumin and heparin injections**. Mean ± SD of the plasma FFA concentrations. A. Rats were injected (intravenous) with either albumin or saline. B. Rats were injected (intraperitoneal) with heparin. N = 4 in each group. The effects of the albumin injection were assessed within the NAR albumin group with a one-way repeated ANOVA. A two-way repeated ANOVA was used for the heparin study. §*P *≤ 0.05 vs. pre-injection values within the NAR group. * *P *≤ 0.05 vs. the SDR group at the same time point.

Data from early *in vitro *studies with purified LPL [20,27] showed that albumin allows lipolysis to proceed by acting as a FFA acceptor. Here, we show that an intravascular injection of albumin into NAR elicited an almost linear increase in the plasma FFA levels over time (Figure 2A). NAR plasma FFA levels increased 4-fold from a baseline of 0.36 ± 0.05 to nearly the control levels of 1.34 ± 0.16 mEq/L 90 min after the injection (*P *≤ 0.05) (Figure 2A). SDR and NAR groups were also injected with saline to control for possible changes in plasma volume, which actually did not cause significant fluctuations in plasma FFA levels (Figure 2A). A heparin injection, which is known to increase the plasma activity of LPL [28], promptly increased (*P *≤ 0.05) the plasma FFA levels in NAR but had no effect on SDR (Figure 2B). The highest increase in plasma FFA levels in NAR was observed 30 min after the heparin injection (from a baseline of 0.52 ± 0.12 to 1.40 ± 0.41 mEq/L, *P *≤ 0.05) and decreased thereafter (Figure 2B). Despite the lack of albumin acting as a FFA acceptor in NAR, the heparin injection was effective in acutely increasing plasma FFA levels, probably because lipoproteins can carry an additional load of FFA [29]. The lack of an observable effect of heparin on SDR's plasma FFA levels is probably due to low concentrations of substrate (TG-rich lipoproteins) after the long fasting period.

**Figure 3:**
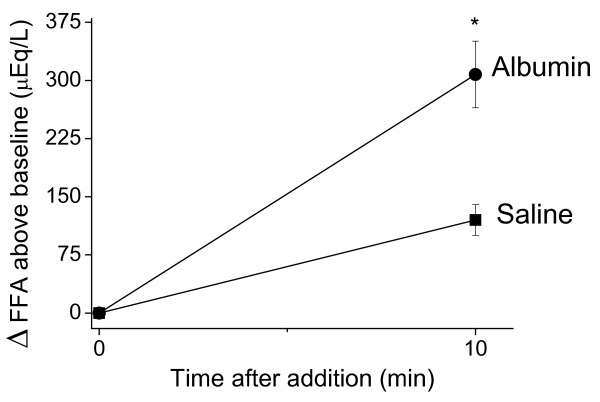
**Exogenous albumin stimulates *in vitro *free fatty acid (FFA) release into fresh NAR serum**. NAR serum was incubated at 37°C in the presence of exogenous albumin or vehicle (saline). Immediately after these additions and after 10 min of incubation, aliquots were taken for FFA analysis. Data are mean ± SD of the change in FFA concentration above the baseline value. N = 3 and the samples were incubated in duplicate. * *P *≤ 0.05 vs. saline at the same time point.

The effect of exogenous albumin (final concentration of 30 mg/mL) on *in vitro *lipolysis was also studied. The addition of albumin to NAR serum promoted a nearly 3-fold stimulation in the amount of released FFA (Figure 3). The extent of lipolysis, taken as FFA accumulation above the initial values in NAR serum, increased (*P *≤ 0.05) from 120.3 ± 21.1 (saline control) to 310.2 ± 51.5 uEq/L in the presence of exogenous albumin.

Since female NAR present with more prominent abnormalities in lipid metabolism compared to male NAR [12,30], we also investigated whether intravenous albumin injection would stimulate plasma lipolysis in fasted male NAR. Similarly to female NAR, plasma lipolysis was stimulated in male NAR as indicated by a 2-fold increase (*P *≤ 0.05) in plasma free glycerol levels 90 min after the intravenous injection of albumin (from a pre-injection level of 125.0 ± 17.7 to 260.4 ± 31.3 umol/L at the 90^th ^min after the injection).

## Discussion

Our results indicate that hypertriglyceridemia in analbuminemic rats does not occur as a result of increased hepatic triglyceride synthesis and output (Figure 1). Consequently, a slower removal rate of the plasma triglycerides seems to delineate the origin of NAR hypertriglyceridemia. Triglyceride removal from the circulation by targeted tissues occurs after their breakdown into glycerol and FFA [31]. Most of the FFA (~ 80%) that are derived from the LPL-mediated lipolysis of plasma triglyceride-rich lipoproteins are mixed within the pool of albumin-bound FFA before their transport into tissues [23,32]. Our data show that plasma lipolysis is stimulated by the exogenous administration of albumin in NAR, both *in vivo *(Figure 2A) and *in vitro *(Figure 3). These data support that the lack of albumin limits LPL activity and thus, slows down the removal rate of plasma triglyceride-rich lipoproteins.

Regarding the contribution of hepatic triglyceride production our findings differ from previous studies that reported this process is enhanced in fasted female NAR [13] and in glucose-fed male NAR [17]. However, in sucrose-fed NAR groups, our results are in accordance with data from Joles et al. [9] that showed similar liver lipogenesis rates between fed NAR and controls. Some discrepancies may be due to important differences in study design. First, we paired the rat groups by age (3-month old) and not by body mass as did Catanozzi et al. [17]. It is noteworthy that NAR are lighter than SDR at the same age. Second, the time of the day at which the experiments were conducted must be considered, since circadian rhythms are well known modifiers of sterol and lipid biosynthesis. While we performed the metabolic studies in the morning from 10:00 to 13:00 h, Catanozi et al. [17] evaluated TG secretion rates from 17:00 to 19:00 h. Third, fasting period and/or type of diet prior to the experiments could also interfere in the results. Thus, based on our findings one can conclude that, either during fasting or fed states, hepatic triglyceride production does not contribute to NAR hypertriglyceridemia. However, since we measured liver lipogenesis only, we may not exclude that extra-hepatic lipogenesis may contribute to NAR hyperlipidemia as previously shown by Joles et al. [9].

A plasma FFA deficit (Table 1) has long been described in NAR [14] and is also observed in analbuminemic humans [7]. However, its physiological basis has never been experimentally addressed. Our attempts to stimulate plasma lipolysis, either by releasing the hypothesized lipolysis inhibition (albumin injection) or by increasing plasma LPL activity (heparin injection), caused a significant increase in plasma FFA levels in NAR, although only albumin induced a virtually complete correction of the NAR plasma FFA deficit (Figure 2A). The fast rise in plasma FFA levels induced by the injection of heparin in NAR (Figure 2B) reveals that the restraint on intravascular lipolysis is not due to defective LPL activity. Therefore, the metabolic machinery involved in plasma triglyceride breakdown and FFA release is not severely compromised in NAR and the plasma FFA deficit reflects an impaired lipolysis due mainly to a lack of plasma albumin as an acceptor. However, since lower LPL activity was reported in NAR parametrial adipose and heart tissues [10,19], it is possible that the lower tissue-specific LPL may constitute another restraint for FFA flux into the NAR tissues [23].

Previous *in vitro *enzymatic studies using purified LPL clearly demonstrated that albumin acts as a FFA acceptor and allows lipolysis to proceed [20,27]. Yet, this fundamental role of albumin in the control of lipolysis was not known to operate *in vivo*. The present results (Figure 2A) expand these previous *in vitro *findings of albumin playing a permissive role in intravascular lipolysis. To avoid any misinterpretation between albumin having a FFA sparing effect or stimulating lipolysis, we used an *in vitro *setup to assess the effects of exogenous albumin on the NAR serum lipolysis rate (Figure 3). This result clearly indicates that albumin is affecting lipolysis, i.e. stimulating the appearance of FFA in NAR plasma.

In conclusion, although NAR present with marked liver hypertrophy, the hepatic triglyceride synthesis and output rates do not contribute to the analbuminemic hypertriglyceridemia. Instead, an impaired triglyceride removal from the circulation may cause this alteration. It is proposed that a lack of plasma albumin to act as a FFA acceptor inhibits the intravascular lipolysis rate, which in turn hampers plasma triglyceride breakdown and its plasma removal.

## Competing interests

The authors declare that they have no competing interests.

## Authors' contributions

**TRF **designed some experiments, conducted the experiments and the assays, analyzed the data, and wrote the manuscript. **AEV **designed the study, critically discussed the results, proposed new experiments, and critically reviewed the final version of the manuscript. **HCFO **designed the experiments, discussed the experimental protocols, discussed the data, and wrote the manuscript. All authors have read and approved the final version of this manuscript.
